# The Warmth of Sarudango: Modelling the Huddling Behaviour of Japanese Macaques (*Macaca fuscata*)

**DOI:** 10.3390/ani14233468

**Published:** 2024-12-01

**Authors:** Cédric Sueur, Shintaro Ishizuka, Yu Kaigaishi, Shinya Yamamoto

**Affiliations:** 1 Université de Strasbourg, IPHC UMR7178, CNRS, 67000 Strasbourg, France; 2 Institut Universitaire de France, 75005 Paris, France; 3 Faculty of Life Science and Biotechnology, Fukuyama University, Fukuyama 729-0292, Japan; ishizuka.shintaro.0206@gmail.com; 4 Wildlife Research Center, Kyoto University, Kyoto 606-8501, Japan; kaigaraseki@gmail.com (Y.K.); shinyayamamoto1981@gmail.com (S.Y.); 5 Kyoto University Institute for Advanced Study, Kyoto 606-8501, Japan

**Keywords:** thermoregulation, individual-based model, computational ethology, primatology, self-organisation

## Abstract

Huddling behaviour is observed across various mammalian and avian species. Huddling, a behaviour wherein animals maintain close physical contact with conspecifics for warmth and social bonding, is widely documented among species in cold environments as a crucial thermoregulatory mechanism. Interestingly, on Shodoshima, Japanese macaques form exceptionally large huddling clusters, often exceeding 50 individuals, a significant deviation from the smaller groups observed in other populations (Arashyama, Katsuyama, and Taksakiyama) and climates. This study aims to uncover the mechanisms behind the formation and size of these huddling clusters, proposing that such behaviours can be explained by simple probabilistic rules influenced by environmental conditions, the current cluster size, and individual decisions. Employing a computational model developed in Netlogo, we seek to demonstrate how emergent properties like the formation and dissolution of clusters arise from collective individual actions. We investigate whether the observed differences in huddling behaviour, particularly the larger cluster sizes on Shodoshima compared to those in colder habitats, reflect variations in social tolerance and cohesion. The model incorporates factors such as environmental temperature, cluster size, and individual decision-making, offering insights into the adaptability of social behaviours under environmental pressures. The findings suggest that temperature plays a crucial role in influencing huddling behaviour, with larger clusters forming in colder climates as individuals seek warmth. However, the study also highlights the importance of joining and leaving a cluster in terms of probability in the dynamics of huddling behaviour. We discussed the large clusters on Shodoshima as a result of a combination of environmental factors and a unique social tolerance and cohesion among the macaques. This study contributes to our understanding of complex social phenomena through the lens of self-organisation, illustrating how simple local interactions can give rise to intricate social structures and behaviours.

## Simple Summary

Japanese macaques, also known as snow monkeys, are known for their ability to huddle together in cold weather to stay warm. However, the size of these huddling groups can vary greatly across different regions, with exceptionally large clusters observed on Shodoshima Island compared to smaller groups in colder areas. This study used computer simulations to understand the factors influencing these huddling behaviours. By modelling individual decisions to join or leave a huddle based on temperature, group size, and other simple rules, we found that environmental conditions and local social dynamics play key roles in determining huddle size. Larger groups tend to form more clusters, but individual preferences and social tolerance also shape these behaviours. Our findings highlight how simple behaviours at the individual level can lead to complex group patterns. Understanding these dynamics not only helps us learn more about how animals survive in extreme conditions but also provides insights into the social structures and adaptability of Japanese macaques, which could inform conservation strategies and studies on animal behaviour.

## 1. Introduction

Numerous mammalian and avian species, including rodents [1,2,3], primates [4,5,6,7,8,9], and birds [10,11,12,13], exhibit a behaviour known as huddling [8], where they maintain close physical contact with their conspecifics [14]. From birth, rodent litters demonstrate this instinctively as the dam compiles her pups into a single, warm aggregation. This communal tendency is not only crucial in the early days, evidenced by pups between 2 and 10 postnatal days orienting themselves towards littermates [15], but also plays a significant role in maintaining group integrity as individuals actively seek to return to the cluster’s centre when displaced [1,16]. In colder climates, Emperor penguins utilise huddling as a critical survival strategy, forming large groups to conserve heat during the brutal Antarctic winters, a behaviour that underscores the dynamic nature of these aggregations [11,12]. Similarly, bats huddle together within roosts to maintain warmth during rest periods, showcasing huddling as a widespread thermoregulatory mechanism across species [17]. These examples illustrate that huddling, while serving the primary purpose of thermoregulation, also facilitates complex social interactions within and across species, highlighting its significance in animal behaviour and survival strategies. Huddling also exists in other mountain primates [4,6,7,9,18,19]. For instance, huddling in snub-nosed monkeys [20,21,22], such as Yunnan snub-nosed monkeys (*Rhinopithecus bieti*) and Sichuan snub-nosed monkeys (*Rhinopithecus roxellana*), reflects an adaptive strategy shaped by thermoregulation, predator avoidance, and social organisation. These behaviours are influenced by environmental conditions, with larger and more cohesive huddling clusters, particularly involving females and juveniles, forming during colder nights to conserve heat and reduce predation risks.

**Figure 1:**
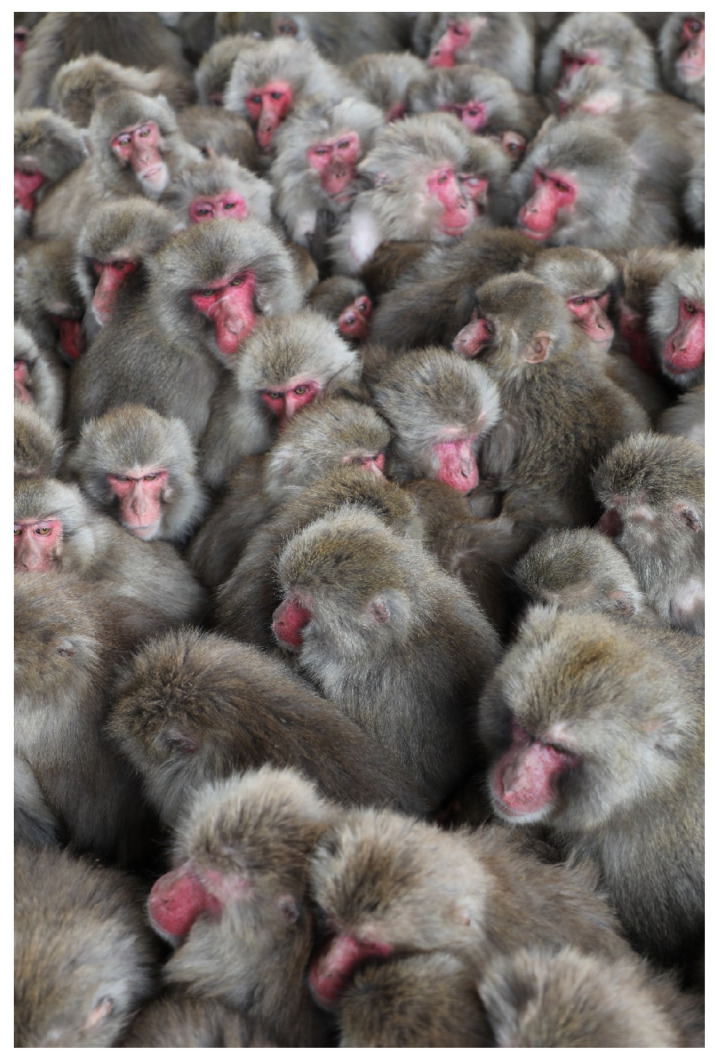
Extra-large huddling cluster (>51 individuals according to Zhang and Watanabe, 2007 [26]), or sarudango, formed in Shodoshima. Crédit: Cédric Sueur.

On Shodoshima Island, located in the southern part of Japan, Japanese macaques (*Macaca fuscata*) [23] exhibit a unique behaviour by forming exceptionally large huddling clusters, often including more than 50 individuals and, in winter, numbers can even surpass 100 [24,25,26] (Figure 1). This phenomenon, characterised by its scale, is exclusive to Shodoshima among Japanese macaques and stands out as a cultural anomaly when compared to the typically smaller cluster sizes observed in other populations of the species across various habitats. Notably, in the warmer climate of Shodoshima Island, huddles reached averages of 17.1 and 15.9 individuals in two observed groups [26]. In contrast, habitats like Shiga Heights experience colder climates where Japanese macaques form clusters rarely exceeding ten individuals, mainly comprising close kin [27]. On average, huddle sizes among Japanese macaques tend to consist of around three individuals, though this number varies slightly across different groups and locations. For instance, the Arashiyama group has an average huddle size of 2.3 individuals [28], the Shiga Heights group averages 3.1 [27], the Minoo group typically sees huddles of exactly three individuals [29], and two groups in Takasakiyama observe larger average sizes of 4.5 and 4.7 individuals [26].

The objective of this study is to investigate the underlying mechanisms that govern the formation and size of huddling clusters among Japanese macaques. Specifically, we aim to examine whether the dynamics of huddling cluster formation and dissolution can be attributed to simple probabilistic rules that are influenced by environmental temperature, the current size of the cluster, and the individual decisions to join or leave a cluster. By presenting a computational model [30,31] that encapsulates these factors, we propose to elucidate how such emergent properties arise from the collective interactions of individuals within these groups [32,33,34,35]. So, our model seeks to answer whether the observed variances in huddling behaviour among different groups of Japanese macaques—such as those on Shodoshima Island compared to groups in colder regions—can be explained through these two local decision-making processes, reflecting in a way the social tolerance and cohesion of Shodoshima macaques. This approach allows us to explore the adaptability of social behaviours to environmental pressures and to understand how seemingly complex social structures and behaviours can emerge from simple, local interactions.

These foundational principles draw parallels with the concept of self-organisation [32,33] seen across a wide range of biological systems, from insect swarms and fish schools to human crowds, and even extend to applications in robotics [36,37]. Self-organisation refers to the process by which pattern and order in a system emerge from local interactions between parts of an initially disordered system without direction from an external source. In the context of biological aggregations, such as those observed in Japanese macaques, self-organised behaviours are crucial for survival, enabling individuals to adapt to environmental challenges through collective action. In insects, for example, self-organisation is evident in the complex structures or the foraging patterns of ants, where individual actions, guided by simple rules like pheromone trails or local environmental cues, result in sophisticated colony-level outcomes [38]. Fish schools utilise a similar mechanism [39], with each fish adjusting its position relative to its neighbours based on simple rules related to distance and alignment, leading to the dynamic, cohesive movement of the entire school. Human crowds, too, exhibit self-organised behaviour, with pedestrian flows forming patterns and adapting to obstacles based on individual decisions influenced by the movement and presence of nearby people [40,41]. In robotics, these principles are harnessed to design autonomous systems capable of complex behaviours through the implementation of simple, local rules. Robots in a swarm can coordinate tasks, navigate environments, and respond to challenges collectively, mirroring the decentralised decision-making processes found in natural systems [36,37].

By exploring these concepts within the framework of huddling behaviours in Japanese macaques, this study seeks to contribute to a broader understanding of how simple behavioural rules can give rise to complex social phenomena. It posits that the emergent properties of huddling, such as the formation and dissolution of clusters in response to environmental stimuli, can be explained through the lens of self-organisation, offering insights into the underlying mechanisms that drive social cohesion and adaptability in animal groups.

## 2. Material and Methods

### 2.1. Studied Subjects

All empirical data referenced in this paper have been previously published [9,26,28], and we have utilised them solely as indicators to demonstrate the accuracy of our model in capturing the huddling behaviour observed in each of the studied groups. The provisioning sites are open areas where the macaques are approximately twice a day. The variation in group size and composition was attributed to maturity, death, or migration. Notably, during food provisioning, most adult females and a few high-ranking males stayed at the provisioning site, while other low-ranking males foraged at peripheral sites. The troops exhibited linear dominance hierarchies among adult females and high-ranking males, which remained consistent throughout each study period [42]. The methods used in the studies across these different locations allowed for consistent scoring of huddling behaviours, including cluster size, the number of clusters, and the timing of joining and leaving events. The sampling strategies enable comparable observations of huddling dynamics.

Arashiyama [28]: The observations were made at the Arashiyama Monkey Park (coordinates: 35.011 N, 135.676 E), Iwatayama, in Kyoto, central Japan. The study focused on the Arashiyama E group of Japanese macaques, which included a mix of adult females, adult males, and their immature offspring. These macaques were free-ranging within the park, located on the eastern slope of Iwatayama Hill, and have been habituated by humans since 1954, with all individuals being identifiable by physical characteristics. The group size and composition varied between 137 adults observed (101 females and 36 males, 26 December 2000 to 29 March 2001) and 125 adults (99 females and 26 males, 26 December 2001 to 29 March 2002). Data were recorded using scan, opportunistic, and focal animal sampling. When macaques formed a huddle, he recorded the size, shape, and composition of the huddle, as well as the posture, body direction, and relative position of each individual in the huddle. Scan sampling involves systematically observing a group of individuals at pre-determined time intervals and recording the behaviours or positions of all visible individuals at the moment of observation. This method provides a snapshot of group dynamics and is particularly useful for studying behaviours that occur frequently or are distributed across the group. Opportunistic sampling refers to the observation and recording of behaviours whenever they are encountered without adhering to a structured time frame or predefined individual selection. This method is useful for capturing rare or unpredictable events that might otherwise be missed in systematic sampling. Focal sampling entails closely observing a single individual for a specified period, recording all occurrences of specific behaviours exhibited by that individual. This approach allows for detailed data collection on an individual’s behaviour and is especially effective for studying activities that are less frequent or highly variable among individuals. In the observed study, the average size of huddles among the Japanese macaques was approximately 2.32 (2–7) individuals. These huddles were more common and correlated with colder temperatures, especially noticeable during afternoons when their habitat lacked direct sunlight. Temperatures ranged from −1.4 °C to 15.8 °C during the observation period.

The group’s description is summarised in Table 1.

Katsuyama [9]: The study focused on a group of free-ranging Japanese macaques located in Katsuyama (35.05 N; 133.42 E), Okayama Prefecture, Japan, with detailed genealogical relationships and individual identification established through characteristic features. The group has been under observation and artificially provisioned since 1958. Between April 2012 and March 2013, the time of observation, the group’s composition was 68 adults (13 males, 55 females). Huddling behaviour was recorded using 30 min focal sampling periods, during which a single individual was observed, and its huddling behaviour was documented, including the number and identities of individuals in the huddle, as well as the start and end times of the behaviour. Temperatures ranged from 2.2 °C to 17.3 °C during the observation period. The mean size of huddles was 2.2 individuals.

Shodoshima [26]: The study was conducted at the Choshikei Monkey Park (34.518 N, 134.243 E) on Shodoshima Island, located in the Inner Sea of Japan, which covers an area of 153.5 km^2^. The island is home to seven natural groups of Japanese macaques, characterised by its deeply cut glens and vertical cliffs. Among these, Groups SA and SB frequently visited the park, spending most of their daytime in the vicinity. The provisioning of the subject group began in 1956, leading to a rapid population increase and the eventual division into the SA and SB groups in the 1960s. Group SA consisted of approximately 454 individuals, with a composition of about 10 adult males, 149 adult females, 21 subadult males, 164 juveniles, 110 infants, and some individuals unidentified. Group SB consisted of approximately 333 individuals, with a composition of about 8 adult males, 120 adult females, 14 subadult males, 113 juveniles, 102 infants, and some individuals unidentified. Zhang and Watanabe (2007) [26] stated that the number of monkeys they counted during each scan sampling was 200 ± 39 or 230 ± 36 in Shodoshima. SA and SB had mean cluster sizes of approximately 3.5 and 3.9, respectively, with clusters typically being ordinary in size. Extra-large clusters (more than 51 individuals) were rare, occurring when temperatures fell below 10 °C. In winter, mean cluster sizes surged to 17.1 for SA and 15.9 for SB, showing a significant increase in clustering behaviour during colder months. The largest cluster sizes recorded were 137 for SA and 115 for SB, with extra-large clusters comprising 7% of the total clusters and including about 30% of the groups’ populations. This pattern indicates a clear tendency for larger clusters during lower temperatures, with winter temperatures averaging around 9 °C.

Takasakiyama [26]: The Takasakiyama Monkey Park (33.258 N, 131.533 E), located on the east coast of Kyushu and about 295 km south of Choshikei Monkey Park, hosts Study Groups TB and TC. Group TB consists of approximately 432 individuals, with a composition of about 21 adult males, 97 adult females, 23 subadult males, 163 juveniles, 102 infants, and some individuals unidentified. Group TC consists of approximately 743 individuals, with a composition of about 25 adult males, 175 adult females, 17 subadult males, 319 juveniles, 193 infants, and some individuals unidentified. Zhang and Watanabe (2007) [26] stated that the number of monkeys they counted during each scan sampling was 251 ± 34 or 306 ± 49 in Takasakiyama. TB and TC showed no formation of large or extra-large clusters in summer, with mean cluster sizes remaining small at about 2.8 for both groups. Winter mean cluster sizes increased slightly to 4.6 for TB and 4.8 for TC, significantly smaller than those of the Shodoshima groups in winter. Extra-large clusters (more than 51) were not observed in the Takasakiyama groups during winter, and large clusters (between 20 and 51 individuals) constituted only about 2% of the total, involving a minor portion of the groups’ populations. Despite similar cold conditions, the clustering behaviour in the Takasakiyama groups did not significantly change with temperature, illustrating a distinct difference in social clustering compared to the Shodoshima groups.

### 2.2. Model Description

*Purpose:* This section describes the model according to the ODD protocol (i.e., overview, design concepts, and details) [43,44]. This theoretical investigation utilises computational simulations in Netlogo 6.4 [45] to align closely with observed behaviours of Japanese macaques, specifically focusing on the phenomenon of sarudango, or the formation of large huddling clusters. The primary aim is to simulate the intricacies of huddling behaviour as a response to cold environmental stimuli and group size, with a keen focus on understanding how individual decisions to join or leave huddles. In our model, we manipulated four independent variables: temperature, group size, the probability of an individual joining a cluster, and the probability of leaving a cluster. We measured two primary outcomes: the number of individuals per cluster and the total number of clusters. Our hypothesis posits that as the temperature and the probability of joining a cluster increase, we would see an increase in the number of individuals per cluster while the total number of clusters would decrease. Conversely, an increase in group size is anticipated, elevating both the number of individuals per cluster and the overall number of clusters. The probability of leaving a cluster is expected to inversely affect both the number of individuals per cluster and the total number of clusters, potentially leading to smaller cluster sizes and a greater number of clusters overall. In the model, once an agent joins a cluster, it becomes stationary within that cluster but continues to assess its internal state and external conditions. The probability of leaving a cluster is dynamically evaluated at each time step based on the leaving threshold and the agent’s perception of discomfort or suboptimal conditions. If the conditions exceed the agent’s tolerance level, as determined by the leaving threshold, it will leave the cluster and resume movement, either to explore or to join another cluster. Field studies suggest that while macaques prioritise thermal benefits in their initial huddling decisions, they may adjust their position or cluster membership in response to social relationships, dominance hierarchies, or environmental changes. The model’s inclusion of a dynamic leaving probability captures this flexibility, allowing agents to transition between clusters as needed.

#### Entities, State Variables, and Scales

- Entities: The model features two main entities: ‘agents’ and ‘patches’. Agents represent individual monkeys and are homogeneous in terms of attributes, lacking differentiation based on age, dominance, or sex. This simplification allows us to focus on the fundamental aspects of huddling behaviour without the added complexity of individual variance. Each agent possesses a set of behavioural rules that govern its decisions to join or leave huddles based on environmental conditions and the proximity of other agents. The simulated environment consists of a grid of 16 × 16 patches that wrap both horizontally and vertically, creating a continuous space for agents to move within. This spatial arrangement ensures that agents at the edges of the environment can interact with those on the opposite edge, mimicking an unbounded natural habitat. The patches themselves are uniform, with no specific features or characteristics that directly influence the agents’ movements or decisions. Instead, the environment acts as a backdrop against which the dynamics of huddling behaviour emerge. The decision to abstract the environment and standardise the agents serves to highlight the emergent properties of huddling as a collective behaviour. By stripping away individual differences and environmental complexities, we aim to distil the essence of huddling dynamics, focusing on how simple rules of interaction can give rise to complex social structures.
- State Variables: For macaques, state variables include cluster-id and huddle-time. ‘cluster-id’ plays a crucial role in our simulation, serving as an identifier for the specific huddle to which an agent belongs. It enables us to track the formation and dissolution of huddles over time, providing insights into the social dynamics within the macaque population. ‘huddle-time’ represents the amount of time (in simulation ticks) an individual has spent within a specific huddle. This variable allows us to analyse patterns of huddle stability and duration, offering a window into the importance of huddles for thermal regulation and social interaction among macaques. Each patch in the environment holds the variable ‘cluster’ to indicate the presence (or absence) of a huddle. A positive value signifies an active huddle, while a value of −1 indicates no huddle presence. This distinction is vital for visualising and understanding the spatial distribution of huddles within the simulated environment.
- Scales: The simulation operates across temporal and spatial dimensions, with each tick symbolising a discrete unit of time and spatial scale abstracted to represent the size of the simulated environment, devoid of direct real-world analogues. Temperature ranges from 0 to 10, simulating varying environmental conditions that influence huddling behaviour. Temperature affects the macaques’ propensity to form huddles, with lower temperatures generally encouraging more frequent and larger huddles for warmth. We explore six distinct group sizes: 70, 100, 130, 160, 300, and 400 individuals, replicating the group sizes of the macaque populations of Arashiyama, Katsuyama, Shodoshima, and Takasakiyama. This range allows us to examine how group size influences the dynamics of huddle formation, maintenance, and the social network’s structure within the simulated population.

### 2.3. Design Concepts

- Emergence: The emergent patterns of huddle formation and dissolution in the model are a direct consequence of individual agents’ decisions, which are influenced by a combination of stochastic elements and deterministic rules. At each time step, agents decide whether to join or leave a cluster based on a comparison between their internal threshold probabilities and a random number.
  The joining threshold (J) is defined as a parameter representing the sensitivity of an individual to join a huddling cluster. It reflects the internal predisposition of an agent to respond to environmental (e.g., temperature) and social cues (e.g., cluster size). Specifically, lower values of J indicate that an individual is more likely to join a cluster, even under less compelling conditions, whereas higher values suggest a more selective or cautious decision-making process for joining. This threshold balances the agent’s need for warmth and social interaction against the inherent risks of crowding or energy expenditure. The probability for an agent to join a cluster (*P_join_*) is influenced by the ambient temperature and the size of the cluster. This probability is calculated as follows, ensuring that the likelihood of joining increases with the cluster size but decreases with higher temperatures, reflecting the natural drive for warmth and sociality. This hypothesis is based on what is observed in the field [26], even if some variation may exist according to social relationships; here, the aim is to make simple rules according to aggregation rules observed in self-organisation processes in such behaviours [46] and such animals [47].

(1) $$P_{join}=\frac{Nci}{Ncmax}\times \frac{1}{T}\times \frac{1}{J}$$

where *J* for join-threshold is a predefined value set by the modeller, ranging from 1 to 10, adjusting the sensitivity of agents to join a cluster. *T* is the temperature. *Nci* denotes the size of the current cluster an individual is considering joining. *Ncmax* signifies the maximum cluster size, equivalent to the overall group size, setting an upper limit on cluster growth.

The leaving threshold (L) is a parameter that quantifies an individual’s sensitivity to discomfort or dissatisfaction within a cluster, prompting the decision to leave. It represents the internal tolerance level of an agent, where lower values imply a greater likelihood of leaving even under minor discomfort, while higher values suggest a strong commitment to staying within the cluster. The parameter accounts for individual variability in the trade-off between thermal benefits and potential social or spatial constraints within a group. Conversely, the probability of leaving (*P_leave_*) is set by the agent based on its personal comfort within the cluster, expressed as follows:

(2) $$P_{leave}=\frac{Ncmax-Nci}{Ncmax}\times \frac{1}{L}$$

where L is the leaving threshold, meaning a constant adjusted by the modeller on the sensitivity of the agent to leave the current cluster. It ranges from 10 to the group size. These thresholds are central to the model as they determine the probabilistic decisions of agents to join or leave a cluster, influencing the dynamics of huddle formation and dissolution. By incorporating stochastic elements, the model mimics the variability seen in natural systems, while the deterministic components ensure alignment with observed behavioural patterns. The joining threshold (J) and leaving threshold (L) were empirically informed values derived from observations of huddling behaviour in macaques. The range for the joining threshold (1 to 10) and leaving threshold (starting at 10) reflects patterns seen in field studies and initial model analyses. These thresholds were chosen to balance simplicity with biological relevance, aligning with the observed behavioural tendencies of macaques in huddling contexts. The joining threshold (J) of 1 to 8 was set to capture variations in individual propensity to join clusters. Lower values represent a high sensitivity to social and environmental cues for joining, while higher values represent more selective joining behaviour. This range aligns with empirical observations indicating that macaques vary in their social dynamics and thermoregulatory needs depending on environmental conditions and individual social bonds. The leaving threshold (L), starting at 10, was selected to represent the minimal discomfort tolerance observed before individuals leave a cluster. This threshold begins at a higher value to reflect the fact that leaving a cluster is generally less frequent than joining, as individuals prioritise maintaining thermal and social benefits until a significant discomfort is perceived. Preliminary analyses showed that values for L below 10 and extreme values for J (e.g., beyond 8) did not align well with field data or yield optimal model fits. The ranked models consistently demonstrated that thresholds outside these ranges were not among the best-performing models. These choices are grounded in empirical results from Shodoshima and other macaque populations, as well as iterative model validation. By testing different values, we confirmed that this parameterisation optimally captures the dynamics of huddle formation and dissolution, ensuring biological plausibility and predictive accuracy in our simulations. This approach highlights how individual thresholds interact with environmental and social factors to drive emergent huddling patterns.

- Sensing: In this model, agents are endowed with the ability to sense not only the ambient temperature but also the proximity of conspecifics within a certain range, which is crucial for making informed decisions about huddling. Specifically, agents can detect other agents within a radius of 2 patches, a feature that simulates their perception of nearby individuals [48].
- Interaction: Huddle formation represents a key interaction mode, where macaques engage in selective positioning and alignment relative to others based on social affinity and environmental considerations. The ‘update-cluster-positions’ section of your model is responsible for organising the spatial distribution of agents within a cluster, ensuring that they are evenly spaced. This function is critical for simulating how individual agents position themselves in relation to others within the same cluster, which can affect their interactions and the overall dynamics of the cluster.
- Stochasticity: This model incorporates stochastic elements to simulate the unpredictability inherent in the agents’ decisions and movements, mirroring the variability observed in natural systems. At each simulation step, a random number ranging between 0 and 1 is generated for each agent [49]. This randomness plays a crucial role in the decision to join a cluster (*P_join_*) and the decision to leave a cluster (*P_leave_*).

#### Details

- Initialisation: The simulation initiates with macaques randomly distributed across the environment, each assigned a default *cluster-id* of −1 to denote absence from any huddle and a *huddle-time* of zero. At each time step, agents assess their surroundings and make decisions based on the join and leave probabilities. These probabilities are dynamically adjusted based on the current environmental temperature and the social context (e.g., the size of nearby clusters). Agents without a cluster engage in exploratory movement, randomly turning, and moving forward. This behaviour allows them to encounter other agents and potentially join new clusters. Once part of a cluster, agents remain stationary until they decide to leave, based on the leaving threshold, either to join another cluster or to remain alone.
- Input: Absent specific external inputs regarding variable environmental conditions or individual macaque traits, the model’s focus narrows to the internal dynamics of huddle formation, predicated on the agents’ interactions and ambient temperature.
- End: A simulation concludes either when all agents have successfully joined a cluster, regardless of the total number of clusters formed, or when the simulation surpasses 20,000 steps. This step limit was established based on observations from preliminary simulations, which indicated that the majority concluded before reaching 20,000 steps. Implementing this cutoff was necessary because, under certain combinations of independent variable values, not all individuals would join a cluster within a reasonable timeframe, necessitating a predefined endpoint to ensure the simulation’s termination.
- Outputs: We conducted measurements of both the number of clusters and the individuals per cluster across each simulation. Simulations were performed for various group sizes (N = 70, 100, 130, 160, 300, and 400), with each group undergoing 100 repetitions for each combination of temperature (from 1 to 10°), joining threshold (from 1 to 10 individuals), and leaving threshold. We realised this using BehaviorSpace in Netlogo [50,51]. BehaviorSpace (Netlogo 6.4) is a software tool integrated with Netlogo that allows you to perform experiments with models. BehaviorSpace runs a model many times, systematically varying the model’s settings and recording the results of each model run. This process is sometimes called ‘parameter sweeping’. It lets you explore the model’s ‘space’ of possible behaviours and determine which combinations of settings cause the behaviours of interest. Specifically, for N = 70, we tested leaving thresholds at intervals of 10, ranging from 10 to 70, resulting in a total of 56,000 simulations. For the other group sizes, we used larger intervals of 40 for the leaving threshold, which resulted in 40,000 simulations per group size. We did this for two reasons: first, to avoid long calculations, and second, because preliminary analyses showed a weak influence of leaving threshold on huddling cluster formation. Altogether, this led to a cumulative total of 256,000 simulations.

### 2.4. Statistical Analyses

The independent variables in our study included temperature, joining threshold, leaving threshold, and group size. Given the violation of the normality and homoscedasticity assumptions that underpin the standard linear regression framework, and considering that the dependent variables represent count data, we opted for a Generalised Linear Model (GLM) employing negative binomial regression for both dependent variables: the number of clusters and the number of individuals per cluster. The Theta values and their standard errors were examined, confirming the appropriateness of using negative binomial regression as opposed to a Poisson model. Furthermore, the log-likelihood values for both models indicate a satisfactory fit to the data, demonstrating the effectiveness of the negative binomial regression in this context.

Interactions between independent variables initially resulted in elevated Variance Inflation Factors (VIFs), prompting their removal to mitigate multicollinearity [52]. Subsequent analysis confirmed that the VIF values for all predictors fell well below the threshold of 5, indicating minimal multicollinearity and ensuring the reliability of the model’s estimates. This adjustment underscores our model predictors’ independence and the robustness of our findings. Furthermore, comparative analyses between the GLM with a Poisson distribution and the Linear Model (LM) with a Gaussian distribution, with and without interactions, consistently identified the same factors as influential across comparable models. This consistency reinforces the robustness of our results.

Model selection [53] utilised the Akaike Information Criterion (AIC) and the Bayesian Information Criterion (BIC) through the stepAIC function from the MASS package [54]. This process iteratively evaluated various models to strike an optimal balance between model complexity and fit quality. Both criteria favoured a model that included all four predictors, affirming their significant role in explaining the variability observed in the number of clusters and the number of individuals per cluster. An in-depth examination of the selected model’s coefficients revealed significant associations between the dependent variables and the predictors.

For a comprehensive visualisation of our model’s insights, we generated plots showcasing the estimated coefficients and their confidence intervals. These plots, crafted using ggplot2 [55], not only supplement our numerical analysis but also facilitate an intuitive understanding of the predictive relationships. By visually depicting the influence and precision of each predictor, we offer a clear and accessible interpretation of how each independent variable affects the number of clusters and the number of individuals per cluster, enhancing the interpretability of our statistical findings.

Comparison with empirical data: In our study, we aimed to elucidate the impact of temperature on the social clustering behaviour of primates across various locations by quantifying the number of individuals per cluster as a function of temperature. To achieve this, we crafted linear equations for each site based on descriptions of studied subjects and sites. By coding this information, we could obtain a linear regression for empirical data to compare with theoretical data: Arashiyama, where we observed a decrement from 3 individuals per cluster at 1 degree to none at 10 degrees, modelled as *y* = −0.33*x* + 3.33; Katsuyama, exhibiting a constant 2.2 individuals regardless of temperature, hence *y* = 2.2; Takasakiyama, which demonstrated a reduction from 4.8 to 2.8 individuals across the temperature range, captured by *y* = −0.22*x* + 4.8; and Shodoshima, showing a decrease from 30 to 20 individuals, represented as *y* = −1.11*x* + 31.11. Concerning simulations, we determined the slope (a) by dividing the change in individuals per cluster by the temperature range, thereby quantifying the rate of change in cluster size with temperature. The intercept (b) was subsequently derived from the linear equation *y* = *ax* + *b*, leveraging the initial cluster size at the lowest observed temperature. This analytical approach facilitated a comprehensive understanding of the temperature-dependent dynamics in primate social clustering, revealing significant variances in how different locations respond to thermal changes.

**Figure 2:**
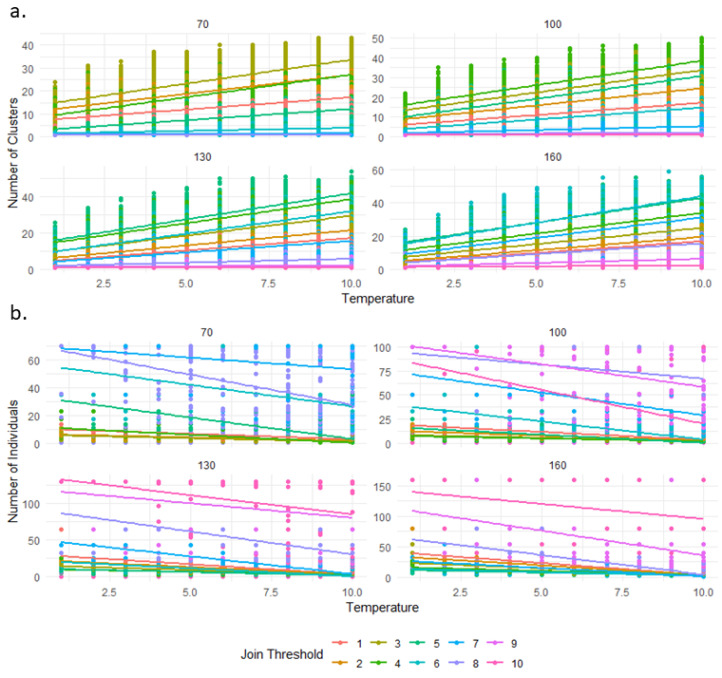
(**a**) Number of clusters and (**b**) number of individuals per cluster as a function of temperature across different group sizes, with each joining threshold distinguished by colour. Each point is a simulation, and several simulations may overlap. The data points are plotted directly on the graph to show the raw distribution of individual clusters, while linear regression lines indicate the overall trend within each join-threshold category. Plots separate the data by group size (number above each plot), allowing for a clear comparison of how the relationship between temperature and cluster size varies across groups.

**Figure 3:**
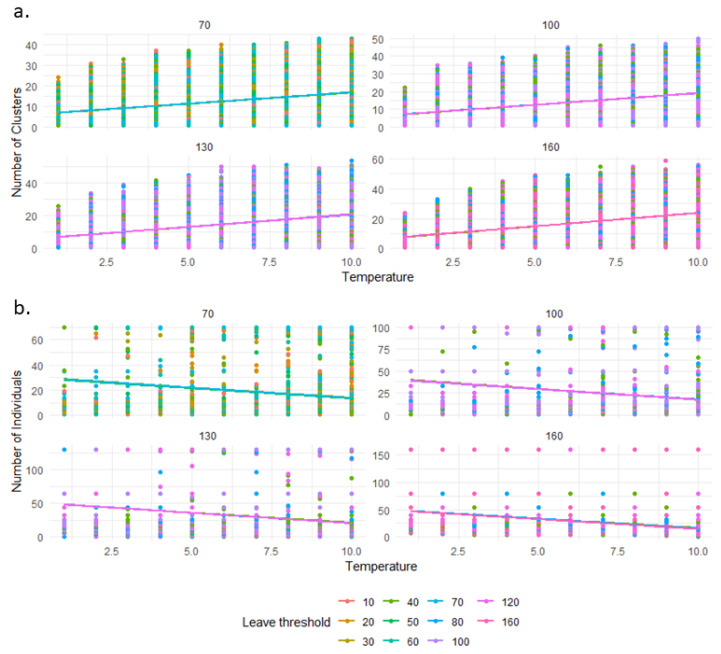
(**a**) Number of clusters and (**b**) number of individuals per cluster as a function of temperature across different group sizes, with each leaving threshold distinguished by colour. Each point is a simulation, and several simulations may overlap. The data points are plotted directly on the graph to show the raw distribution of individual clusters, while linear regression lines indicate the overall trend within each join-threshold category. Plots separate the data by group size (number above each plot), allowing for a clear comparison of how the relationship between temperature and cluster size varies across groups.

Models with group sizes of 300 and 400 individuals were excluded from the statistical analyses because they produced simulated cluster sizes exceeding the largest observed cluster size in Shodoshima (>137 individuals) by 9% and 14.5%, respectively, compared to only 0.3% for N = 160. These discrepancies suggest that the processes driving huddling behaviour and cluster formation in macaques operate at a smaller scale than the entire group size, likely involving interactions within local substructures such as families or nearby individuals. Therefore, to maintain the biological relevance of the model and reduce unnecessary variables, we excluded the 300- and 400-individual group sizes from further analysis. It is important to note that the Shodoshima population itself was not excluded from the analyses. Instead, the model was adjusted to better align with observed data from Shodoshima and other sites, as detailed in Table 1 and Figure 2 and Figure 3. All other group sizes and study sites, including those from Shodoshima, were retained to ensure a comprehensive comparison across populations. This decision reflects our focus on modelling huddling dynamics within empirically observed constraints and highlights the importance of subgroup-level interactions in shaping cluster sizes.

Utilising the lm function in R, we constructed linear models for each unique combination of group size and join-threshold within our dataset. For each model, we calculated the absolute difference between the empirical intercept (the y-intercept derived from the Linear Model) and the corresponding theoretical intercept. We did the same with the slopes. Then, we ranked the models according to the differences between empirical data and theoretical data, from the lowest to the highest discrepancy.

All analyses were conducted in Rstudio [56,57] with R and α = 0.05.

Netlogo code, scripts, data, and supplementary materials are available at Zenodo: https://doi.org/10.5281/zenodo.11233016.

## 3. Results

### 3.1. Number of Clusters

**Figure 4:**
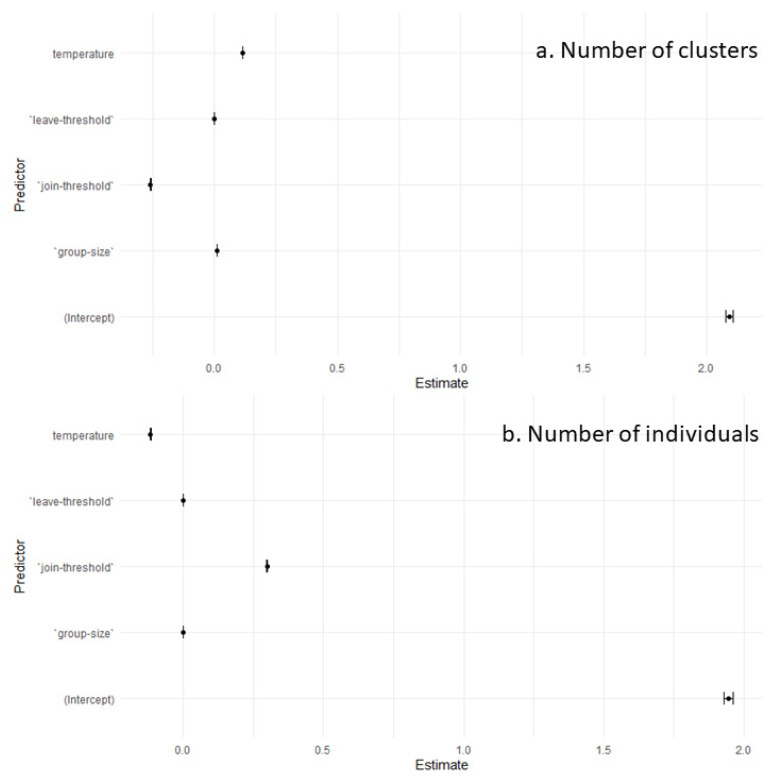
The figure depicts the estimated regression coefficients and their 95% confidence intervals from a Generalised Linear Model (GLM) for (**a**) the number of clusters and (**b**) the number of individuals per cluster. The y-axis represents the predictors, including the model’s intercept, join-threshold, leave-threshold, temperature, and group size. The x-axis shows the estimated coefficients reflecting the expected change in the response variable for a one-unit change in each predictor. Points indicate the magnitude of each estimate, while the bars denote the range of the confidence intervals. A horizontal line at zero would indicate no effect. This visual summary complements the numerical output, illustrating the impact and uncertainty of each predictor on the response variable.

The model fitted to our data comprising 165,851 degrees of freedom (165,847 residual degrees of freedom) revealed a substantial improvement from the null model, as indicated by a reduction in deviance from 281,187 for the null model to 170,645 for the residual. This improvement reflects the significant explanatory power of our predictors. In our GLM analysis, the number of clusters was found to be negatively influenced by the joining threshold, as indicated by a coefficient estimate of −0.259 (*p* < 0.00001, Figure 2a and Figure S1a), suggesting that an increase in the joining threshold is associated with a decrease in the number of clusters. Conversely, the leave-threshold, with a coefficient estimate of 0.000142 (*p* = 0.011, Figure 3a and Figure S2a), and the temperature, with an estimate of 0.1145 (*p* < 0.00001), both showed a positive relationship, indicating that increases in these variables are associated with an increase in the number of clusters. The group size also had a positive impact on the number of clusters, with an estimate of 0.00861 (*p* < 0.00001), suggesting that larger group sizes are linked to a higher number of clusters. These significant *p*-values indicate a strong level of confidence in these relationships (Table 1, Figure 4a).

### 3.2. Number of Individuals per Cluster

On 176,000 simulations, 165,853 (5.76%) did not result in any cluster, meaning that we used only 165853 data points for this GLM. This absence of cluster formation is a combination of high group size with a high joining threshold and high temperature. In the Generalised Linear Model analysis for the number of individuals per cluster, the data included 165,851 degrees of freedom (165,847 residual) with an initially high null deviance of 385,500, which was substantially reduced to 174,400 in the residual deviance by including our predictors. This marked reduction in deviance illustrates the predictors’ significant capacity to explain the variability in the number of individuals per cluster. Regarding the influence of the predictors, the joining threshold presented a positive coefficient estimate of 0.294 (*p* < 0.00001, Figure 2b and Figure S1b), indicating that as the joining threshold increases, there is a corresponding rise in the number of individuals per cluster. On the other hand, both the leaving threshold and temperature had negative associations with the dependent variable, evidenced by estimates of −0.00014 (*p* = 0.0019, Figure 3b and Figure S2b) and −0.16 (*p* < 0.00001), respectively; an increase in these predictors leads to a reduction in the number of individuals per cluster. Furthermore, the group size was also found to negatively affect the number of individuals per cluster, with an estimate of −0.00123 (*p* < 0.00001), suggesting that larger groups tend to have fewer individuals per cluster, aligning with intuitive expectations, as the finite size of the group limits the total number of clusters that can form while maintaining an equilibrium in individual distribution. The substantial z-values coupled with extremely low *p*-values confirm the statistical significance of these findings (Table 2, Figure 4b).

### 3.3. Comparison with Empirical Data Distributions

All model fittings are detailed in the Supplementary Materials (Table S1). For the Arashiyama, Katsuyama, and Takasakiyama groups, we observed consistent results with the two top models that best fit the empirical data: the group size was 70, with joining thresholds at 2 and 3, achieving a ranking of 1.5 based on 38 comparisons. For these three groups, the models with the least fit also showed similarities, indicating a group size of 160 and joining thresholds of 9 and 10.

Regarding the Shodoshima group, the four highest-performing models were closely ranked, albeit with minor variations: group sizes were 70, 100, 130, and 160; joining thresholds were 2, 3, 4, and 6, with an overall ranking of 8.5 from 38 comparisons. Conversely, the four models that were the least compatible with the Shodoshima data indicated group sizes of 100, 130, 160, and 160 and joining thresholds of 9, 10, 10, and 10. The combination of a group size of 70 with a joining threshold of 4 naturally precludes the formation of clusters with more than 70 individuals. This is inconsistent with the observations made on Shodoshima, and it is noteworthy that the proportion of significantly larger clusters (those with more than 51 individuals) stands at a mere 0.5%. Similarly, a group size of 100 coupled with a joining threshold of 2 also fails to produce clusters exceeding 100 individuals, deviating from the Shodoshima data, with the proportion of considerably larger clusters being 0%. In contrast, the combination featuring a group size of 130 and a joining threshold of 3 does result in clusters surpassing 100 individuals, aligning with the Shodoshima findings; however, the proportion of exceptionally large clusters is near 0%. Meanwhile, the configuration with a group size of 160 and a joining threshold of 6 generates clusters with over 100 individuals, which corresponds well with the Shodoshima observations, and about 5% of these clusters are significantly larger. Given this analysis, we can infer that the latter combination (joining threshold = 6, group size = 160) offers the closest match to the huddling behaviour observed on Shodoshima.

## 4. Discussion

In this theoretical study, we modelled huddling behaviour in different groups of macaques using simple rules with four variables linked by simple rules. Understanding behavioural mechanisms in primates and other species through modelling and self-organisation principles is crucial for deciphering the complex social dynamics within these groups [47,48,58,59,60]. Computational models and simulations illuminate various aspects of primate behaviour, such as grooming [61], fission [44,62], cohesion [34,63], social transmission [64], and the emergence of complex social networks [65]. These models demonstrate that simple local interactions and behavioural rules can lead to the emergence of complex social patterns observed in primate societies and beyond.

The four key parameters incorporated into our model significantly influence the huddling behaviour of Japanese macaques, affecting both the number of clusters formed and the number of individuals per cluster. Specifically, ambient temperature and the threshold for joining a huddle exhibit a pronounced and more substantial impact on this behaviour compared to the threshold for leaving a huddle and the overall group size. This analysis elucidates the variations in huddling behaviour observed across different groups of Japanese macaques. Temperature plays a critical role in influencing huddling behaviour [16], consistent with our expectations and the model’s design. As temperatures decrease, the likelihood of an individual joining a cluster increases, serving as a mechanism for warmth among macaques. This effect is particularly strong in Shodoshima, indicating a significant impact of temperature on the formation of huddles, or ‘sarudango,’ in this region [26]. However, in other groups, temperature appears to have a lesser influence on huddle formation, suggesting that it is not the sole factor driving this behaviour. Despite huddling being recognised as a thermoregulatory behaviour, its occurrence varies across regions [9,26,28]. For example, in the colder northern Shimokita region, where one might expect huddling to be more prevalent, macaques may instead engage in other individual thermoregulatory behaviours such as sunbathing [66]. Hot spring bathing exists but only at Jigokudani in the Shiga Heights region [67,68]. This disparity raises intriguing questions about the regional variations in adaptive behaviours among Japanese macaques and the factors influencing the spread and adoption of specific thermoregulatory strategies.

Group size exhibits a positive correlation with both the number of clusters and the number of individuals within each cluster. Interestingly, this correlation is not as pronounced in the theoretical data as it is in the empirical data, where no significant relationship exists between the actual group size and the average cluster size. This discrepancy suggests a potential social or cultural influence on huddling dynamics. Modelling the actual observed group sizes, particularly for locations like Shodoshima and Takasakiyama, would predict extraordinarily large clusters of 200 to 400 individuals, which have not been observed in reality. These findings underscore the significant role of social relationships in huddling behaviour [9,24,26,28]. Previous studies support this conclusion, demonstrating that huddling, especially among individuals in direct contact [24], is influenced by grooming relationships and kinship [9]. This evidence points to the complex interplay between social bonds and physical proximity in shaping the huddling patterns of Japanese macaques.

The likelihood of an individual leaving a cluster emerges as the least influential factor on both the number of clusters and the number of individuals per cluster. Despite its relatively minor role, this aspect remains crucial for the dynamics of huddling behaviour. The mechanisms of joining and leaving clusters are fundamental to these dynamics, as they allow individuals the flexibility to move between clusters, potentially joining larger ones. This process of leaving, although not a dominant factor, facilitates the fluidity of huddle formation and dissolution without significantly interacting with group size or temperature. Notably, our model diverges from the pattern of linking the probability of leaving a cluster to external factors such as temperature or cluster size—a distinction from the probability of joining a cluster. Nevertheless, this omission does not hinder the model’s effectiveness in simulating the dynamics of huddling behaviour accurately. While there are no explicit reports of macaques leaving clusters due to overheating—a phenomenon observed in other species like penguins—it presents an intriguing area for further empirical and theoretical investigation. Exploring how such a link might influence huddling behaviour could enhance our understanding of the complex interplay between environmental conditions and social dynamics in shaping these group behaviours.

The most significant factor influencing huddling behaviour among Japanese macaques is the probability of an individual joining a cluster. This mechanism, as demonstrated in Figure 2 and Figure 3 and as implemented in the model, interacts closely with temperature and, indirectly, with group size through the mediation of cluster size. Importantly, this parameter has a distinct value critical for determining the size of ‘sarudango’ at each observed site, which accounts for the observed differences between various groups, particularly between the Shodoshima troops and other groups. The threshold for joining a cluster that yields simulations closely mirroring the situation on Shodoshima is 6, compared to 2 or 3 for other sites. Notably, even though the threshold is only two to three times higher, it leads to the formation of clusters with an average size of 30 to 35 individuals, which is approximately ten times larger than those observed in other groups. This phenomenon resembles self-organisation [32,33], where local rules give rise to complex patterns not readily explained by simple mechanisms alone. Furthermore, these differences cannot be adequately explained by factors other than those already described in the literature, pointing to a cultural divergence between the Shodoshima troops and others. The classification of Japanese macaques within the despotic spectrum typically indicates a social system marked by lower tolerance, higher aggression, and greater inter-individual distances [69,70,71]. Yet, the large rest clusters observed on Shodoshima Island constitute a remarkable deviation, suggesting a level of tolerance, cohesion, and social adaptability not typically expected in a species known for despotic behaviours [72,73,74]. This unique social tolerance and cohesiveness in Shodoshima, akin to some traits seen in groups on Awajishima [75], are crucial for the formation of these clusters. This indicates that the decision to join a huddle may not be solely dictated by individual needs for thermoregulation but also influenced by local social norms and ecological pressures. The case of Shodoshima’s macaques demonstrates that, despite a general inclination towards despotic social structures within the species, variations driven by local ecological conditions and historical group dynamics do exist [72,74,76,77]. Our study highlights how stronger cultural cohesion, as illustrated by the joining threshold, along with a self-organised amplification process, explains the unusually large clusters in Shodoshima and the differences from other sites. This insight underscores the complex interplay between cultural factors and biological behaviours, contributing to our understanding of the variability within social structures of Japanese macaques. While the despotic-tolerant dichotomy has been a useful tool for comparative studies, researchers of Japanese macaques have highlighted that this framework may oversimplify the nuanced social dynamics observed within populations [67,68,69]. Factors such as group history, ecological conditions, and cultural practices likely play a significant role in shaping these behaviours, as evidenced by the contrast between Shodoshima and other groups. Our findings emphasise the flexibility within this spectrum, underscoring the importance of integrating ecological and cultural perspectives into discussions of social structure.

The self-organisation process in huddling behaviour refers to the emergence of complex patterns from simple rules followed by individuals without a centralised control mechanism [32,33]. In the context of Japanese macaques, this process can be understood through the individual decision-making rules about when to join or leave a cluster based on environmental conditions (such as temperature) and social interactions (such as proximity to kin or familiar individuals). The outcome of these decisions at the individual level leads to the formation of large, cohesive clusters observed in specific sites like Shodoshima, which differ significantly in size and composition from those in other locations. This phenomenon of self-organisation is grounded in the principles of complex systems, where local interactions among components (in this case, the macaques) give rise to global patterns (the size and structure of huddling clusters) that cannot be predicted merely by analysing the components in isolation. The model implemented in the study captures these dynamics by incorporating variables such as the probability of joining a cluster, temperature, and group size, demonstrating how variations in these parameters can lead to substantial differences in huddling behaviour across different macaque populations. While our study primarily focused on broader group-level factors such as overall group size, temperature, and joining and leaving thresholds, the potential influence of family size on huddling behaviour is an intriguing consideration. In Japanese macaques, kinship relationships often play a significant role in social interactions, including grooming and physical proximity within huddles [9,19]. Larger family units could potentially form the core of huddling clusters, contributing to both cluster size and cohesion. However, explicitly incorporating family size as a predictor would require detailed genealogical data for each population, which was beyond the scope of our current modelling approach. Preliminary observations suggest that family size may indirectly influence huddling behaviour by shaping social bonds and individual preferences for proximity within clusters. Future research integrating family structure and kinship data could provide deeper insights into the interplay between social relationships and huddling dynamics. Such an approach would enhance our understanding of the micro-level mechanisms driving cluster formation and variability across populations.

We did not include the distinction between island and mainland study sites as a variable in the model, as this factor is confounded with the specific study sites. Katsuyama and Arashiyama are located on the mainland, while Shodoshima is a small island, similar to Awajishima. However, the potential influence of insularity on social tolerance and cultural behaviour in these populations is an intriguing question. Some researchers have hypothesised that the higher social tolerance observed in Shodoshima and Awajishima macaques may be partially driven by the ecological and social constraints associated with island environments. These constraints could foster stronger social cohesion, reduced inter-individual competition, and the emergence of cultural behaviours, such as the formation of large huddling clusters.

The model we devised for this study, while insightful, has its limitations due to its anonymised approach. Specifically, the probability of an individual joining or leaving a cluster is determined solely by the number of individuals present within each cluster (i.e., anonymous or allelimimetism), without accounting for individual identities and social relationships (i.e., selective mimetism) [47,49]. This simplification overlooks the significant impact that social relationships, kinship ties, dominance hierarchies, and individual identities have on huddling behaviour [24]. It is important to clarify that this limitation does not invalidate the model or undermine its viability [78,79,80,81]. Rather, it suggests that individual identity, while not essential for replicating cluster size and dynamics at a macro level, could enhance our understanding of these processes. Incorporating the identities and unique characteristics of macaques into our model represents a promising avenue for future research. However, such an endeavour would necessitate acquiring detailed empirical social networks for all troops covered in this study, along with precise information on the identities of individuals engaged in huddling behaviour dynamics. This includes tracking each individual’s movements into and out of clusters and their interactions at any given moment. Implementing individual identities within the model presents a considerable challenge, not only in terms of model complexity but also in gathering the requisite data in the field with such large groups. Advancements in technology are bringing us ever closer to overcoming these hurdles. Automatic identification of individuals [82] and their behaviours [83] through artificial intelligence offers a path forward, potentially enabling more nuanced and accurate simulations of huddling behaviour that account for the complex interplay of social dynamics and individual preferences. This next step would significantly enrich our understanding of the mechanisms underpinning huddling behaviour and the social fabric of macaque societies, offering deeper insights into the behavioural ecology of these primates.

To conclude, our findings underscore the utility of computational models in shedding light on the intricate social dynamics of animals, revealing how simple local interactions can give rise to the complex social patterns observed in nature. Our model could be applied to huddling behaviour in other primate or animal species. Our findings on Japanese macaques provide an opportunity to compare huddling behaviour with that observed in other mountain primates, such as snub-nosed monkeys, including Yunnan snub-nosed monkeys and Sichuan snub-nosed monkeys [20,21,22]. In both taxa, huddling functions as an adaptive strategy for thermoregulation, predator avoidance, and social bonding, influenced by environmental conditions. However, the average cluster size in snub-nosed monkeys is approximately two individuals, a pattern also observed in Japanese macaques at Katsuyama (mean = 2.2 individuals) and Arashiyama (mean = 2.3 individuals). These similarities suggest a comparable baseline for huddling behaviour across these primates in terms of group size. What sets our study apart is the unique behaviour observed in Japanese macaques on Shodoshima Island, where clusters regularly exceed 50 individuals and sometimes surpass 100 during colder months. These exceptionally large clusters are not observed in snub-nosed monkeys or other Japanese macaque groups. Our computational model suggests that while environmental factors like temperature play a critical role in huddle formation, social dynamics such as increased social tolerance and cohesion among Shodoshima macaques amplify cluster sizes to an extraordinary degree. The observed differences underscore the importance of social structure and ecological context. While snub-nosed monkeys and Japanese macaques at Katsuyama and Arashiyama form small, kin-centred clusters under similar environmental pressures, the larger clusters in Shodoshima reflect a distinct deviation in social organisation. This deviation may arise from cultural factors and enhanced social tolerance unique to the Shodoshima population, which is atypical for the despotic social systems generally described in Japanese macaques. This study not only advances our understanding of primate social patterns but also highlights the potential for further nuanced investigations into the biological and cultural underpinnings of animal behaviours.

## Figures and Tables

**Table 1:** Summary of Japanese macaque study groups, including their geographic locations, coordinates, group sizes, demographic compositions, mean huddle sizes, and temperature ranges during the study periods. Data represent observations from four study sites: Arashiyama, Katsuyama, Shodoshima (SA and SB groups), and Takasakiyama (TB and TC groups).

Group Name	Latitude, Longitude	Group Size	Composition	Mean Huddle Size	Temperature Range (°C)
Arashiyama	35.011 N, 135.676 E	137 (2000–2001), 125 (2001–2002)	101 females, 36 males	2.32	−1.4 to 15.8
Katsuyama	35.05 N, 133.42 E	68	55 females, 13 males	2.2	2.2 to 17.3
Shodoshima SA	34.518 N, 134.243 E	454	10 adult males, 149 adult females, 21 subadult males, 164 juveniles, 110 infants	17.1	9 (winter average)
Shodoshima SB	34.518 N, 134.243 E	333	8 adult males, 120 adult females, 14 subadult males, 113 juveniles, 102 infants	15.9	9 (winter average)
Takasakiyama TB	33.258 N, 131.533 E	432	21 adult males, 97 adult females, 23 subadult males, 163 juveniles, 102 infants	4.6	Varied
Takasakiyama TC	33.258 N, 131.533 E	743	25 adult males, 175 adult females, 17 subadult males, 319 juveniles, 193 infants	4.8	Varied

**Table 2:** The table presents the results of two separate Generalised Linear Models (GLMs) with Poisson distribution: one for the number of clusters and the other for the number of individuals per cluster. Each model includes estimates of the effect sizes (regression coefficients), their standard errors, z-values, and associated *p*-values for the intercept and four predictors: join-threshold, leave-threshold, temperature, and group size. Positive coefficients indicate a positive effect on the dependent variable, whereas negative coefficients suggest a negative effect. The *p*-values test the null hypothesis that each coefficient is zero, with values less than 0.05 indicating statistical significance.

	*Number of Clusters*				*Number of Individuals Per Cluster*			
	Estimate	Std. Error	z Value	Pr (>|z|)	Estimate	Std. Error	z Value	Pr (>|z|)
*(Intercept)*	2.09 × 10^+00^	7.66 × 10^−03^	273.493	<0.00001	1.95 × 10^+00^	8.23 × 10^−03^	236.183	<0.00001
*Join-threshold*	−2.60 × 10^−01^	7.32 × 10^−04^	−3.55 × 10^+02^	<0.00001	2.99 × 10^−01^	7.84 × 10^−04^	381.941	<0.00001
*Leave-threshold*	1.42 × 10^−04^	5.61 × 10^−05^	2.54 × 10^+00^	0.0111	−1.42 × 10^−04^	6.07 × 10^−05^	−2.333	0.0196
*Temperature*	1.15 × 10^−01^	6.52 × 10^−04^	1.75 × 10^+02^	<0.00001	−1.17 × 10^−01^	7.03 × 10^−04^	−165.858	<0.00001
*Group size*	8.62 × 10^−03^	6.49 × 10^−05^	1.33 × 10^+02^	<0.00001	1.23 × 10^−03^	7.05 × 10^−05^	17.504	<0.00001

## Data Availability

The datasets generated and/or analysed during the current study, along with the codes and scripts, are available in the Zenodo repository: https://doi.org/10.5281/zenodo.11233016.

## Supplementary Materials

The following supporting information can be downloaded at https://www.mdpi.com/article/10.3390/ani14233468/s1, Figure S1: a. Number of clusters and b. number of individuals per cluster as a function of join threshold across different group sizes, with each temperature distinguished by colour. Figure S2: a. Number of clusters and b. number of individuals per cluster as a function of Leave threshold across different group sizes, with each temperature distinguished by colour. Table S1: Models of join threshold or leave threshold with group size best fitting in term of slope differences and intercept the empirical data of each site.

## References

[1] Alberts J.R. (1978). Huddling by Rat Pups: Group Behavioral Mechanisms of Temperature Regulation and Energy Conservation. J. Comp. Physiol. Psychol..

[2] Haim A., Van Aarde R., Skinner J. (1992). Burrowing and Huddling in Newborn Porcupine: The Effect on Thermoregulation. Physiol. Behav..

[3] Hayes J.P., Speakman J.R., Racey P.A. (1992). The Contributions of Local Heating and Reducing Exposed Surface Area to the Energetic Benefits of Huddling by Short-Tailed Field Voles (*Microtus agrestis*). Physiol. Zool..

[4] Behnke T. (2012). To Huddle or Not to Huddle: That Is the Question. A Brief Study of the Basis for Huddling Behavior in Eulemur Rubriventer. Indep. Study Proj. (ISP) Collect..

[5] Gestich C.C., Caselli C.B., Setz E.Z. (2014). Behavioural Thermoregulation in a Small Neotropical Primate. Ethology.

[6] Kelley E.A., Jablonski N.G., Chaplin G., Sussman R.W., Kamilar J.M. (2016). Behavioral Thermoregulation in Lemur Catta: The Significance of Sunning and Huddling Behaviors. Am. J. Primatol..

[7] Ogawa H., Takahashi H. (2003). Triadic Positions of Tibetan Macaques Huddling at a Sleeping Site. Int. J. Primatol..

[8] Schradin C., Ancel A. (2022). Huddling. Encyclopedia of Animal Cognition and Behavior.

[9] Ueno M., Nakamichi M. (2018). Grooming Facilitates Huddling Formation in Japanese Macaques. Behav. Ecol. Sociobiol..

[10] Bourne A.R., Soravia C. (2023). Huddling in the Heat? Rarely Seen Thermoregulatory Behaviours as Southern Pied Babblers Turdoides Bicolor Compete for Cool Microsites. Ostrich.

[11] Gilbert C., Robertson G., Le Maho Y., Naito Y., Ancel A. (2006). Huddling Behavior in Emperor Penguins: Dynamics of Huddling. Physiol. Behav..

[12] Gilbert C., Robertson G., Le Maho Y., Ancel A. (2008). How Do Weather Conditions Affect the Huddling Behaviour of Emperor Penguins?. Polar Biol..

[13] Hafez E. (1964). Behavioral Thermoregulation in Mammals and Birds: A Review. Int. J. Biometeorol..

[14] Gilbert C., McCafferty D., Le Maho Y., Martrette J., Giroud S., Blanc S., Ancel A. (2010). One for All and All for One: The Energetic Benefits of Huddling in Endotherms. Biol. Rev..

[15] Grant R.A., Sperber A.L., Prescott T.J. (2012). The Role of Orienting in Vibrissal Touch Sensing. Front. Behav. Neurosci..

[16] Canals M., Bozinovic F. (2011). Huddling Behavior as Critical Phase Transition Triggered by Low Temperatures. Complexity.

[17] Kerth G., Perony N., Schweitzer F. (2011). Bats Are Able to Maintain Long-Term Social Relationships despite the High Fission–Fusion Dynamics of Their Groups. Proc. R. Soc. B.

[18] Eppley T.M., Watzek J., Dausmann K.H., Ganzhorn J.U., Donati G. (2017). Huddling Is More Important than Rest Site Selection for Thermoregulation in Southern Bamboo Lemurs. Anim. Behav..

[19] Chen J., Yang P., Zhang Q., Li W., Wang X., Li J. (2024). Collective Decision-Making in Nocturnal Huddling Sleep: The Influence of Social Factors on Fans and Fandom in Tibetan Macaques. Glob. Ecol. Conserv..

[20] Kirkpatrick R.C., Long Y.C., Zhong T., Xiao L. (1998). Social Organization and Range Use in the Yunnan Snub-Nosed Monkey *Rhinopithecus bieti*. Int. J. Primatol..

[21] Zhang P., Li B., Watanabe K., Qi X. (2011). Sleeping Cluster Patterns and Retiring Behaviors during Winter in a Free-Ranging Band of the Sichuan Snub-Nosed Monkey. Primates.

[22] Li D., Ren B., Grueter C.C., Li B., Li M. (2010). Nocturnal Sleeping Habits of the Yunnan Snub-Nosed Monkey in Xiangguqing, China. Am. J. Primatol..

[23] Nakagawa N., Nakamichi M., Sugiura H. (2010). The Japanese Macaques.

[24] Ishizuka S. (2021). Do Dominant Monkeys Gain More Warmth? Number of Physical Contacts and Spatial Positions in Huddles for Male Japanese Macaques in Relation to Dominance Rank. Behav. Process..

[25] Yamada M. (1966). Five Natural Troops of Japanese Monkeys in Shodoshima Island: I. Distribution and Social Organization. Primates.

[26] Zhang P., Watanabe K. (2007). Extra-Large Cluster Formation by Japanese Macaques (*Macaca fuscata*) on Shodoshima Island, Central Japan, and Related Factors. Am. J. Primatol..

[27] Wada K. (1985). Sleeping Site Cluster of Japanese Macaques in Shiga A1 Troop: About Inter-Individual Relationships. The Study of Spatial Distribution of Japanese Macaque Troops in Shiga Heights.

[28] Ogawa H., Wada K. (2011). Shape of, and Body Direction in, Huddles of Japanese Macaques (*Macaca fuscata*) in Arashiyama, Japan. Primates.

[29] Yasuda J. (1996). Cluster Formation at Sleeping Sites in a Free-Ranging Group of Japanese Macaques at Minoo. Prim. Res..

[30] Bryson J.J., Ando Y., Lehmann H. (2007). Agent-Based Modelling as Scientific Method: A Case Study Analysing Primate Social Behaviour. Philos. Trans. R. Soc. B Biol. Sci..

[31] Wilensky U., Rand W. (2015). An Introduction to Agent-Based Modeling: Modeling Natural, Social, and Engineered Complex Systems with NetLogo.

[32] Camazine S., Deneubourg J.-L., Franks N.R., Sneyd J., Theraula G., Bonabeau E. (2003). Self-Organization in Biological Systems.

[33] Couzin I.D., Krause J. (2003). Self-Organization and Collective Behavior in Vertebrates.

[34] Sueur C., King A.J., Pelé M., Petit O., Gilbert T., Kirkilionis M., Nicolis G. (2013). Fast and Accurate Decisions as a Result of Scale-Free Network Properties in Two Primate Species. Proceedings of the European Conference on Complex Systems 2012.

[35] Sueur C., MacIntosh A.J.J., Jacobs A.T., Watanabe K., Petit O. (2013). Predicting Leadership Using Nutrient Requirements and Dominance Rank of Group Members. Behav. Ecol. Sociobiol..

[36] Dorigo M., Theraulaz G., Trianni V. (2020). Reflections on the Future of Swarm Robotics. Sci. Robot..

[37] Pfeifer R., Lungarella M., Iida F. (2007). Self-Organization, Embodiment, and Biologically Inspired Robotics. Science.

[38] Bonabeau E., Theraulaz G., Deneubourg J.-L., Aron S., Camazine S. (1997). Self-Organization in Social Insects. Trends Ecol. Evol..

[39] Lopez U., Gautrais J., Couzin I.D., Theraulaz G. (2012). From Behavioural Analyses to Models of Collective Motion in Fish Schools. Interface Focus.

[40] Faria J.J., Dyer J.R.G., Tosh C.R., Krause J. (2010). Leadership and Social Information Use in Human Crowds. Anim. Behav..

[41] Helbing D., Johansson A., Al-Abideen H.Z. (2007). Dynamics of Crowd Disasters: An Empirical Study. Phys. Rev. E.

[42] Koyama N. (1967). On Dominance Rank and Kinship of a Wild Japanese Monkey Troop in Arashiyama. Primates.

[43] Grimm V., Berger U., DeAngelis D.L., Polhill J.G., Giske J., Railsback S.F. (2010). The ODD Protocol: A Review and First Update. Ecol. Model..

[44] Sueur C., Maire A. (2014). Modelling Animal Group Fission Using Social Network Dynamics. PLoS ONE.

[45] Tisue S., Wilensky U. NetLogo: A Simple Environment for Modeling Complexity. Proceedings of the International Conference on Complex Systems.

[46] Ancel A., Gilbert C., Poulin N., Beaulieu M., Thierry B. (2015). New Insights into the Huddling Dynamics of Emperor Penguins. Anim. Behav..

[47] Sueur C., Deneubourg J.-L. (2011). Self-Organization in Primates: Understanding the Rules Underlying Collective Movements. Int. J. Primatol..

[48] Puga-Gonzalez I., Sueur C. (2017). Emergence of Complex Social Networks from Spatial Structure and Rules of Thumb: A Modelling Approach. Ecol. Complex..

[49] Sueur C., Petit O., Deneubourg J. (2009). Selective Mimetism at Departure in Collective Movements of Macaca Tonkeana: An Experimental and Theoretical Approach. Anim. Behav..

[50] Lytinen S.L., Railsback S.F. The Evolution of Agent-Based Simulation Platforms: A Review of NetLogo 5.0 and ReLogo. Proceedings of the Fourth International Symposium on Agent-Based Modeling and Simulation.

[51] Railsback S., Ayllón D., Berger U., Grimm V., Lytinen S., Sheppard C., Thiele J.C. (2017). Improving Execution Speed of Models Implemented in NetLogo. J. Artif. Soc. Soc. Simul..

[52] Franke G.R. (2010). Multicollinearity. Wiley International Encyclopedia of Marketing.

[53] Burnham K.P., Anderson D.R. (2004). Multimodel Inference Understanding AIC and BIC in Model Selection. Sociol. Methods Res..

[54] Ripley B., Venables B., Bates D.M., Hornik K., Gebhardt A., Firth D., Ripley M.B. (2013). Package ‘Mass’. Cran R.

[55] Wickham H., Chang W., Wickham M.H. (2016). Package ‘Ggplot2.’ Create elegant data visualisations using the grammar of graphics. Version.

[56] Allaire J. (2012). RStudio: Integrated Development Environment for R. Boston MA.

[57] Racine J.S. (2012). RStudio: A Platform-Independent IDE for R and Sweave. J. Appl. Econom..

[58] Hemelrijk C.K. (2005). Self-Organisation and Evolution of Biological and Social Systems.

[59] Hemelrijk C.K. (2005). A Process-Oriented Approach to the Social Behaviour of Primates. Self-Organisation and Evolution of Social Systems.

[60] Puga-Gonzalez I., Ostner J., Schülke O., Sosa S., Thierry B., Sueur C. (2018). Mechanisms of Reciprocity and Diversity in Social Networks: A Modeling and Comparative Approach. Behav. Ecol..

[61] Puga-Gonzales I., Ostner J., Schülke O., Sosa S., Thierry B., Sueur C. (2017). Proximate Mechanisms Underlying Primates’ Complex Social Networks: A Modelling and Comparative Approach. Folia Primatol..

[62] Sueur C., Deneubourg J.-L., Petit O., Couzin I.D. (2011). Group Size, Grooming and Fission in Primates: A Modeling Approach Based on Group Structure. J. Theor. Biol..

[63] Lehmann J., Korstjens A.H., Dunbar R.I. (2007). Group Size, Grooming and Social Cohesion in Primates. Anim. Behav..

[64] Romano V., Duboscq J., Sarabian C., Thomas E., Sueur C., MacIntosh A.J.J. (2016). Modeling Infection Transmission in Primate Networks to Predict Centrality-Based Risk. Am. J. Primatol..

[65] Romano V., Puga-Gonzalez I., MacIntosh A.J., Sueur C. (2024). The Role of Social Attraction and Social Avoidance in Shaping Modular Networks. R. Soc. Open Sci..

[66] Hanya G., Kiyono M., Hayaishi S. (2007). Behavioral Thermoregulation of Wild Japanese Macaques: Comparisons between Two Subpopulations. Am. J. Primatol..

[67] Takeshita R.S.C., Bercovitch F.B., Kinoshita K., Huffman M.A. (2018). Beneficial Effect of Hot Spring Bathing on Stress Levels in Japanese Macaques. Primates.

[68] Zhang P., Watanabe K., Eishi T. (2007). Habitual Hot-spring Bathing by a Group of Japanese Macaques (*Macaca fuscata*) in Their Natural Habitat. Am. J. Primatol..

[69] Matsumura S. (1999). The Evolution of “Egalitarian” and “Despotic” Social Systems Among Macaques. Primates.

[70] Sueur C., Petit O., De Marco A., Jacobs A.T., Watanabe K., Thierry B. (2011). A Comparative Network Analysis of Social Style in Macaques. Anim. Behav..

[71] Thierry B., Singh M., Kaumanns W. (2004). Macaque Societies: A Model for the Study of Social Organization.

[72] Huffman M.A., Nakagawa N., Go Y., Imai H., Tomonaga M., Huffman M.A., Nakagawa N., Go Y., Imai H., Tomonaga M. (2013). Cultural Diversity of Social Behaviors in Japanese Macaques. Monkeys, Apes, and Humans: Primatology in Japan.

[73] Yamada M. (1971). Five Natural Troops of Japanese Monkeys on Shodoshima Island: II. A Comparison of Social Structure. Primates.

[74] Zhang P., Watanabe K. (2014). Intraspecies Variation in Dominance Style of *Macaca fuscata*. Primates.

[75] Kaigaishi Y., Nakamichi M., Yamada K. (2019). High but Not Low Tolerance Populations of Japanese Macaques Solve a Novel Cooperative Task. Primates.

[76] Hanya G., Yamagiwa J., Karczmarski L. (2014). Japanese Macaques: Habitat-Driven Divergence in Social Dynamics. Primates and Cetaceans: Field Research and Conservation of Complex Mammalian Societies.

[77] Ito T., Hayakawa T., Suzuki–Hashido N., Hamada Y., Kurihara Y., Hanya G., Kaneko A., Natsume T., Aisu S., Honda T. (2021). Phylogeographic History of Japanese Macaques. J. Biogeogr..

[78] Sanchez P.J. (2006). As Simple as Possible, but No Simpler: A Gentle Introduction to Simulation Modeling.

[79] Saracci R. (2006). Everything Should Be Made as Simple as Possible but Not Simpler. Int. J. Epidemiol..

[80] van der Vaart E., Johnston A.S., Sibly R.M. (2016). Predicting How Many Animals Will Be Where: How to Build, Calibrate and Evaluate Individual-Based Models. Ecol. Model..

[81] Wilson A.J., Réale D., Clements M.N., Morrissey M.M., Postma E., Walling C.A., Kruuk L.E., Nussey D.H. (2010). An Ecologist’s Guide to the Animal Model. J. Anim. Ecol..

[82] Paulet J., Molina A., Beltzung B., Suzumura T., Yamamoto S., Sueur C. (2023). Deep Learning for Automatic Detection and Facial Recognition in Japanese Macaques: Illuminating Social Networks. arXiv.

[83] Ardoin T., Sueur C. (2024). Automatic Identification of Stone-Handling Behaviour in Japanese Macaques Using LabGym Artificial Intelligence. Primates.

