# Supplementary material for: The Warmth of Sarudango: Modelling the Huddling Behaviour of Japanese Macaques (Macaca fuscata)
Source: Animals (Basel). 2024 Dec 1;14(23):3468. doi: 10.3390/ani14233468 (PMC11640321; doi:10.3390/ani14233468)
Supplement: Supplementary file 1 [file animals-14-03468-s001.zip › animals-3280608-supplementary.pdf]

Supplementary materials

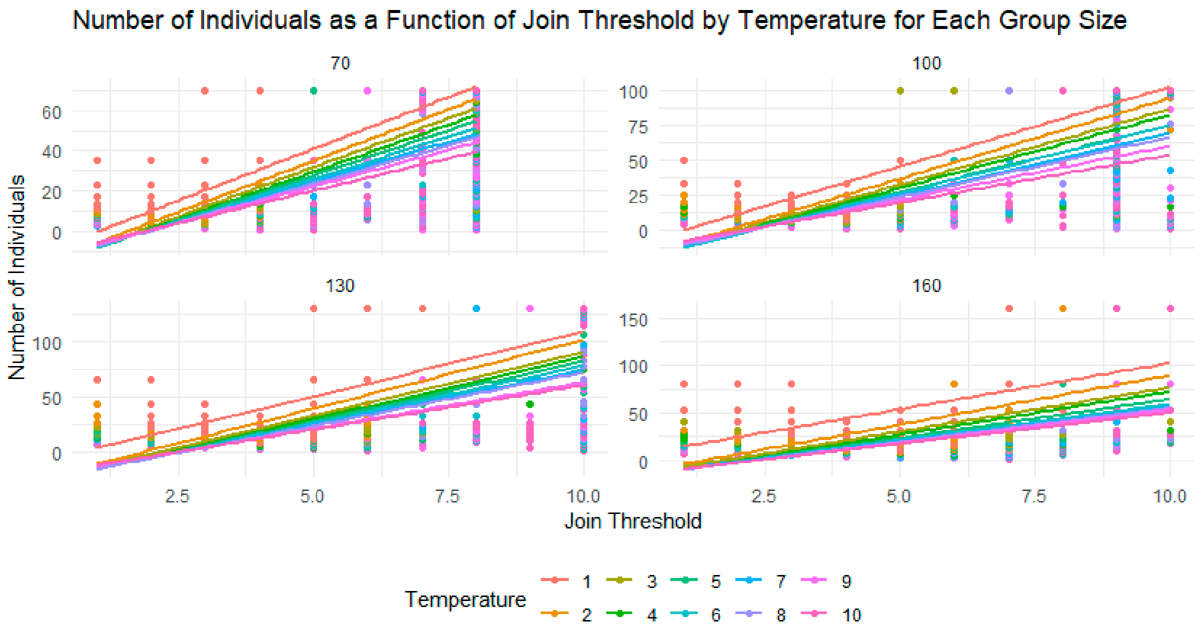

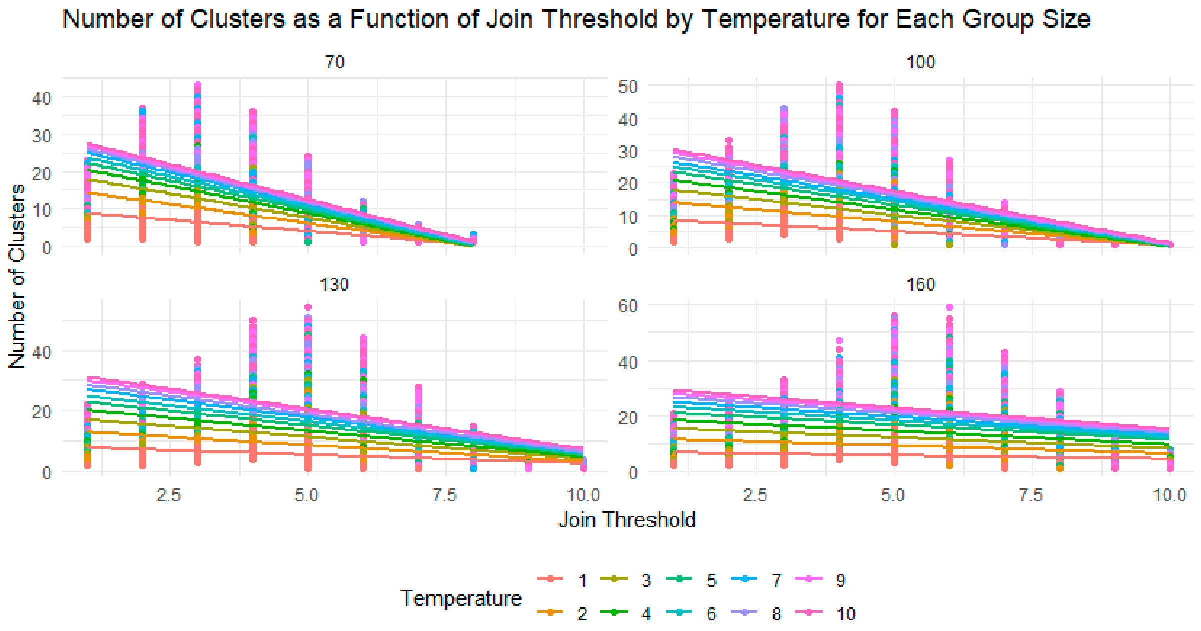

Figure S1: a. Number of clusters and b. number of individuals per cluster as a function of join threshold across different group sizes, with each temperature distinguished by colour.

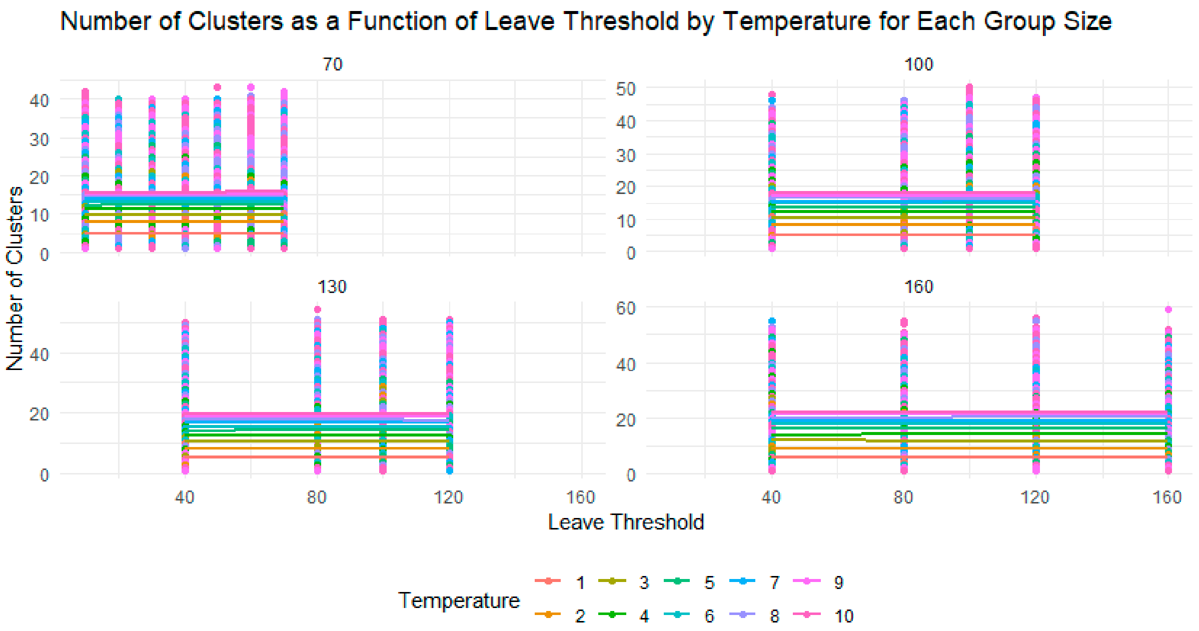

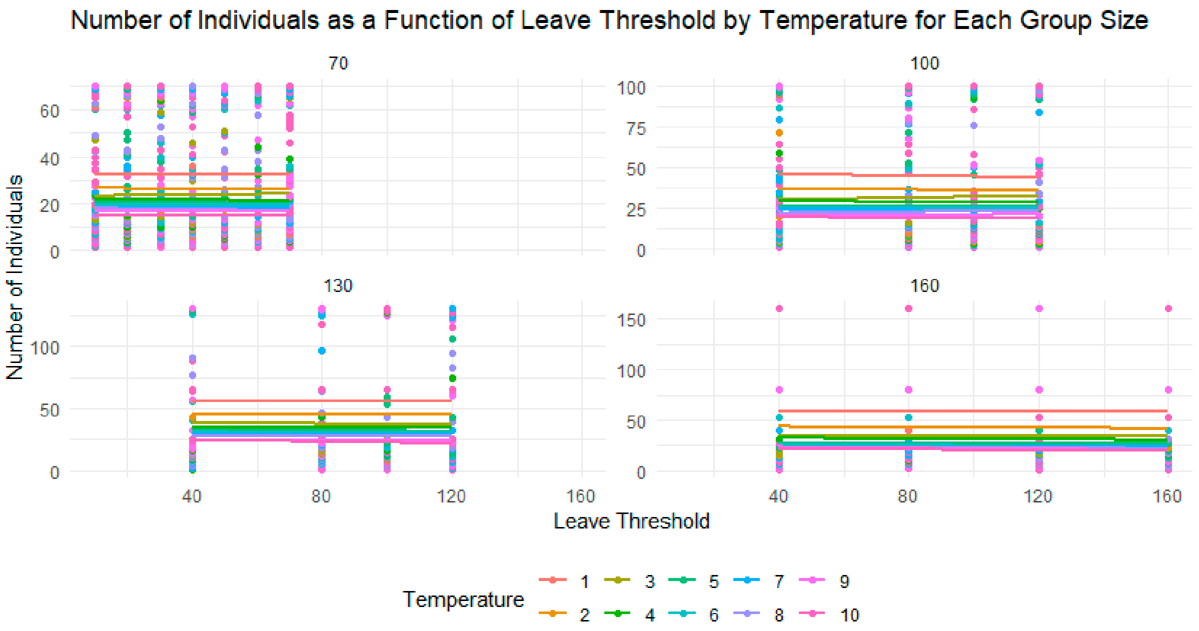

Figure S2: a. Number of clusters and b. number of individuals per cluster as a function of Leave threshold across different group sizes, with each temperature distinguished by colour.

Table S1: Models of join threshold or leave threshold with group size best fitting in term of slope differences and intercept the empirical data of each site.

Shodoshima							
Slope differences				Intercept			
Best fitting model	Group Size	Join threshold	Difference	Best fitting model	Group Size	Leave threshold	Difference
1	70	4	0.05879812	1	130	1	0.4453667
2	100	2	0.06274484	2	70	5	3.1467685
3	160	6	0.0937129	3	160	7	3.1540419
4	130	3	0.1651813	4	160	2	3.4529278
5	160	5	0.18541779	5	160	3	6.5168098
6	130	5	0.20455599	6	130	2	8.2802627
7	160	4	0.25783523	7	130	6	9.2310775
8	130	4	0.29547729	8	100	6	10.2243645
9	70	1	0.32296224	9	100	1	11.1984635
10	100	3	0.42874635	10	160	1	11.3226362
11	100	4	0.50492187	11	100	5	14.0147163
12	70	7	0.54901942	12	160	4	14.8585614
13	100	1	0.55566945	13	130	3	15.9181082
14	100	5	0.57765452	14	160	6	17.3504646
15	70	3	0.61494787	15	100	2	18.0965954
16	70	2	0.61667465	16	70	4	18.9449979
17	130	2	0.88546113	17	160	5	19.4815648
18	130	6	1.03957609	18	70	1	20.2357824
19	160	3	1.05135856	19	130	5	20.3789111
20	160	7	1.60323015	20	130	7	20.7659301
21	130	1	1.66586653	21	130	4	20.7898819
22	100	8	1.77811111	22	100	3	22.3127435
23	70	6	1.97938596	23	100	4	23.3558481
24	70	5	1.98132734	24	70	2	24.2649016
25	160	2	2.04359878	25	70	3	24.8640079
26	100	6	2.65138043	26	70	6	26.5985221
27	130	9	2.83971429	27	160	8	36.9907515
28	160	1	2.85447042	28	70	7	38.9343317
29	70	8	3.22544032	29	70	8	40.1978508
30	100	9	3.569859	30	100	7	44.7939196
31	100	7	3.57832648	31	100	10	59.4548541
32	130	7	3.72981291	32	130	8	61.79706
33	160	10	3.83488709	33	100	8	64.687254
34	130	10	4.19312319	34	100	9	74.0480319
35	130	8	5.1500197	35	160	9	85.5395754
36	160	8	5.33048401	36	130	9	88.747246
37	100	10	5.91495831	37	130	10	106.686452
38	160	9	7.00143506	38	160	10	113.45848

Arashiyama							
Slope differences				Intercept			
Best fitting model	Group Size	Join threshold	Difference	Best fitting model	Group Size	Leave threshold	Difference
1	70	2	0.1633254	1	70	3	2.915992
2	70	3	0.1650521	2	70	2	3.515098
3	100	4	0.2750781	3	100	4	4.424152
4	100	3	0.3512536	4	100	3	5.467256
5	70	1	0.4570378	5	130	4	6.990118
6	130	4	0.4845227	6	130	5	7.401089
7	130	5	0.575444	7	70	1	7.544218
8	160	5	0.5945822	8	160	5	8.298435
9	100	2	0.7172552	9	70	4	8.835002
10	70	4	0.8387981	10	100	2	9.683405
11	160	6	0.8737129	11	160	6	10.429535
12	130	3	0.9451813	12	130	3	11.861892
13	160	4	1.0378352	13	160	4	12.921439
14	70	7	1.3290194	14	100	5	13.765284
15	100	1	1.3356695	15	100	1	16.581537
16	100	5	1.3576545	16	130	6	18.548922
17	130	2	1.6654611	17	130	2	19.499737
18	130	6	1.8195761	18	160	3	21.26319
19	160	3	1.8313586	19	160	7	24.625958
20	160	7	2.3832301	20	130	1	27.334633
21	130	1	2.4458665	21	70	5	30.926768
22	100	8	2.5581111	22	160	2	31.232928
23	70	6	2.759386	23	100	6	38.004364
24	70	5	2.7613273	24	160	1	39.102636
25	160	2	2.8235988	25	130	7	48.54593
26	100	6	3.4313804	26	70	6	54.378522
27	130	9	3.6197143	27	160	8	64.770751
28	160	1	3.6344704	28	70	7	66.714332
29	70	8	4.0054403	29	70	8	67.977851
30	100	9	4.349859	30	100	7	72.57392
31	100	7	4.3583265	31	100	10	87.234854
32	130	7	4.5098129	32	130	8	89.57706
33	160	10	4.6148871	33	100	8	92.467254
34	130	10	4.9731232	34	100	9	101.828032
35	130	8	5.9300197	35	160	9	113.319575
36	160	8	6.110484	36	130	9	116.527246
37	100	10	6.6949583	37	130	10	134.466452
38	160	9	7.7814351	38	160	10	141.23848

Takasakiyama							
Slope differences				Intercept			
Best fitting model	Group Size	Join threshold	Difference	Best fitting model	Group Size	Leave threshold	Difference
1	70	2	0.2733254	1	70	3	1.445992
2	70	3	0.2750521	2	70	2	2.045098
3	100	4	0.3850781	3	100	4	2.954152
4	100	3	0.4612536	4	100	3	3.997256
5	70	1	0.5670378	5	130	4	5.520118
6	130	4	0.5945227	6	130	5	5.931089
7	130	5	0.685444	7	70	1	6.074218
8	160	5	0.7045822	8	160	5	6.828435
9	100	2	0.8272552	9	70	4	7.365002
10	70	4	0.9487981	10	100	2	8.213405
11	160	6	0.9837129	11	160	6	8.959535
12	130	3	1.0551813	12	130	3	10.391892
13	160	4	1.1478352	13	160	4	11.451439
14	70	7	1.4390194	14	100	5	12.295284
15	100	1	1.4456695	15	100	1	15.111537
16	100	5	1.4676545	16	130	6	17.078922
17	130	2	1.7754611	17	130	2	18.029737
18	130	6	1.9295761	18	160	3	19.79319
19	160	3	1.9413586	19	160	7	23.155958
20	160	7	2.4932301	20	130	1	25.864633
21	130	1	2.5558665	21	70	5	29.456768
22	100	8	2.6681111	22	160	2	29.762928
23	70	6	2.869386	23	100	6	36.534364
24	70	5	2.8713273	24	160	1	37.632636
25	160	2	2.9335988	25	130	7	47.07593
26	100	6	3.5413804	26	70	6	52.908522
27	130	9	3.7297143	27	160	8	63.300751
28	160	1	3.7444704	28	70	7	65.244332
29	70	8	4.1154403	29	70	8	66.507851
30	100	9	4.459859	30	100	7	71.10392
31	100	7	4.4683265	31	100	10	85.764854
32	130	7	4.6198129	32	130	8	88.10706
33	160	10	4.7248871	33	100	8	90.997254
34	130	10	5.0831232	34	100	9	100.358032
35	130	8	6.0400197	35	160	9	111.849575
36	160	8	6.220484	36	130	9	115.057246
37	100	10	6.8049583	37	130	10	132.996452
38	160	9	7.8914351	38	160	10	139.76848

Katsuyama							
Slope differences				Intercept			
Best fitting model	Group Size	Join threshold	Difference	Best fitting model	Group Size	Leave threshold	Difference
1	70	2	0.4933254	1	70	3	4.045992
2	70	3	0.4950521	2	70	2	4.645098
3	100	4	0.6050781	3	100	4	5.554152
4	100	3	0.6812536	4	100	3	6.597256
5	70	1	0.7870378	5	130	4	8.120118
6	130	4	0.8145227	6	130	5	8.531089
7	130	5	0.905444	7	70	1	8.674218
8	160	5	0.9245822	8	160	5	9.428435
9	100	2	1.0472552	9	70	4	9.965002
10	70	4	1.1687981	10	100	2	10.813405
11	160	6	1.2037129	11	160	6	11.559535
12	130	3	1.2751813	12	130	3	12.991892
13	160	4	1.3678352	13	160	4	14.051439
14	70	7	1.6590194	14	100	5	14.895284
15	100	1	1.6656695	15	100	1	17.711537
16	100	5	1.6876545	16	130	6	19.678922
17	130	2	1.9954611	17	130	2	20.629737
18	130	6	2.1495761	18	160	3	22.39319
19	160	3	2.1613586	19	160	7	25.755958
20	160	7	2.7132301	20	130	1	28.464633
21	130	1	2.7758665	21	70	5	32.056768
22	100	8	2.8881111	22	160	2	32.362928
23	70	6	3.089386	23	100	6	39.134364
24	70	5	3.0913273	24	160	1	40.232636
25	160	2	3.1535988	25	130	7	49.67593
26	100	6	3.7613804	26	70	6	55.508522
27	130	9	3.9497143	27	160	8	65.900751
28	160	1	3.9644704	28	70	7	67.844332
29	70	8	4.3354403	29	70	8	69.107851
30	100	9	4.679859	30	100	7	73.70392
31	100	7	4.6883265	31	100	10	88.364854
32	130	7	4.8398129	32	130	8	90.70706
33	160	10	4.9448871	33	100	8	93.597254
34	130	10	5.3031232	34	100	9	102.958032
35	130	8	6.2600197	35	160	9	114.449575
36	160	8	6.440484	36	130	9	117.657246
37	100	10	7.0249583	37	130	10	135.596452
38	160	9	8.1114351	38	160	10	142.36848
